# Supplementary material for: A Versatile Suspended Lipid Membrane System for Probing Membrane Remodeling and Disruption
Source: Membranes (Basel). 2022 Nov 25;12(12):1190. doi: 10.3390/membranes12121190 (PMC9784602; doi:10.3390/membranes12121190)
Supplement: Supplementary file 1 [file membranes-12-01190-s001.zip › membranes-1923921-supplementary.pdf]

A Versatile Suspended Lipid Membrane System for Probing Membrane Remodeling and Disruption

Achinta Sannigrahi*, Vishwesh Haricharan Rai‡, Muhsin Vannan Chalil‡, Debayani Chakraborty‡, Subrat Kumar Meher, Rahul Roy*

Department of Chemical Engineering, Indian Institute of Science, Bangalore 560012, Karnataka, India

* Email of correspondence: sannigrahiachinta@gmail.cohwum, rahulroy@iisc.ac.in

‡ Equal contribution.

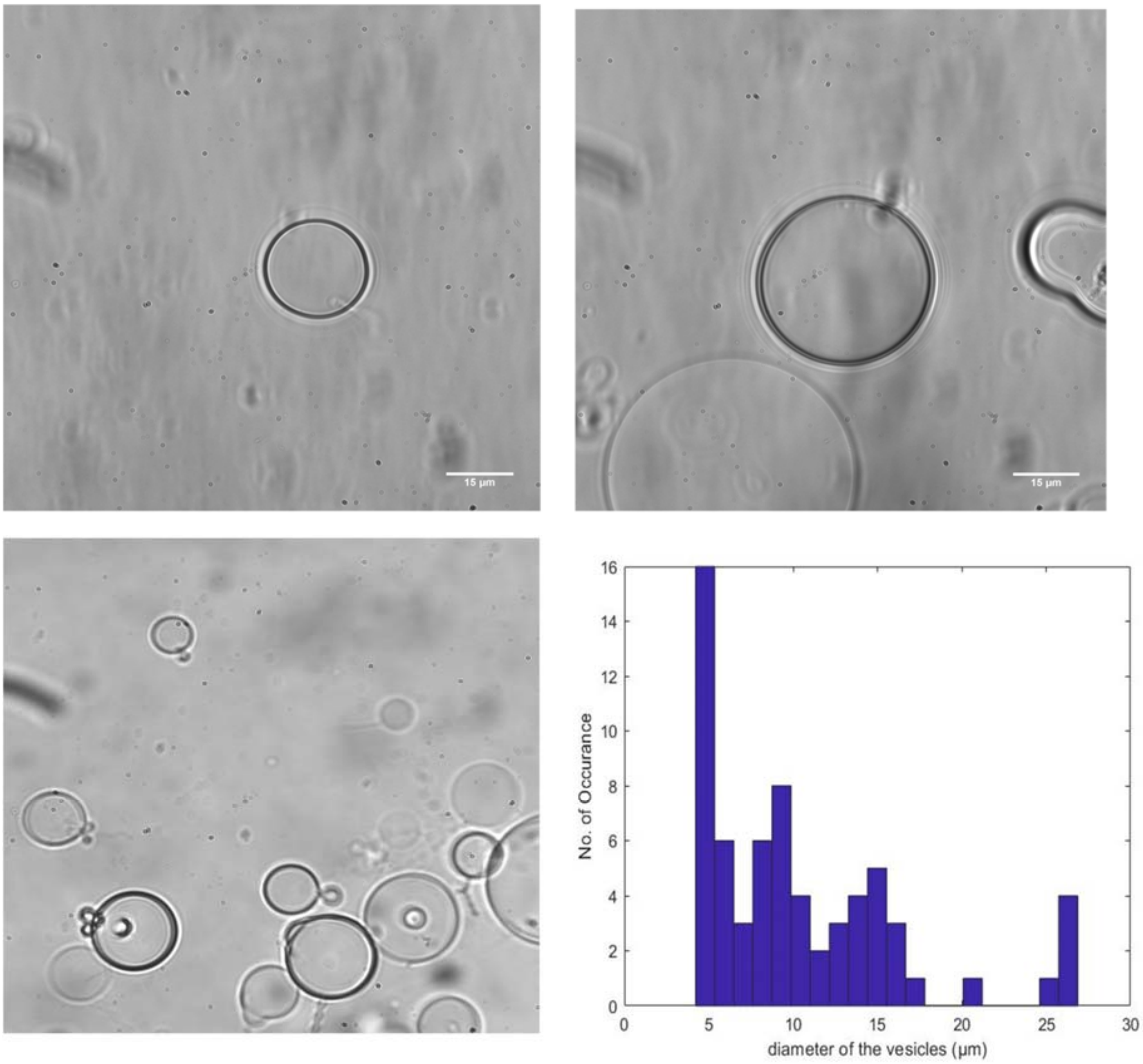

Figure S1: Representative phase contrast image of GUVs (POPC + 30% Chol). Size distribution of the GUVs formed by the gel assisted GUV formation method.

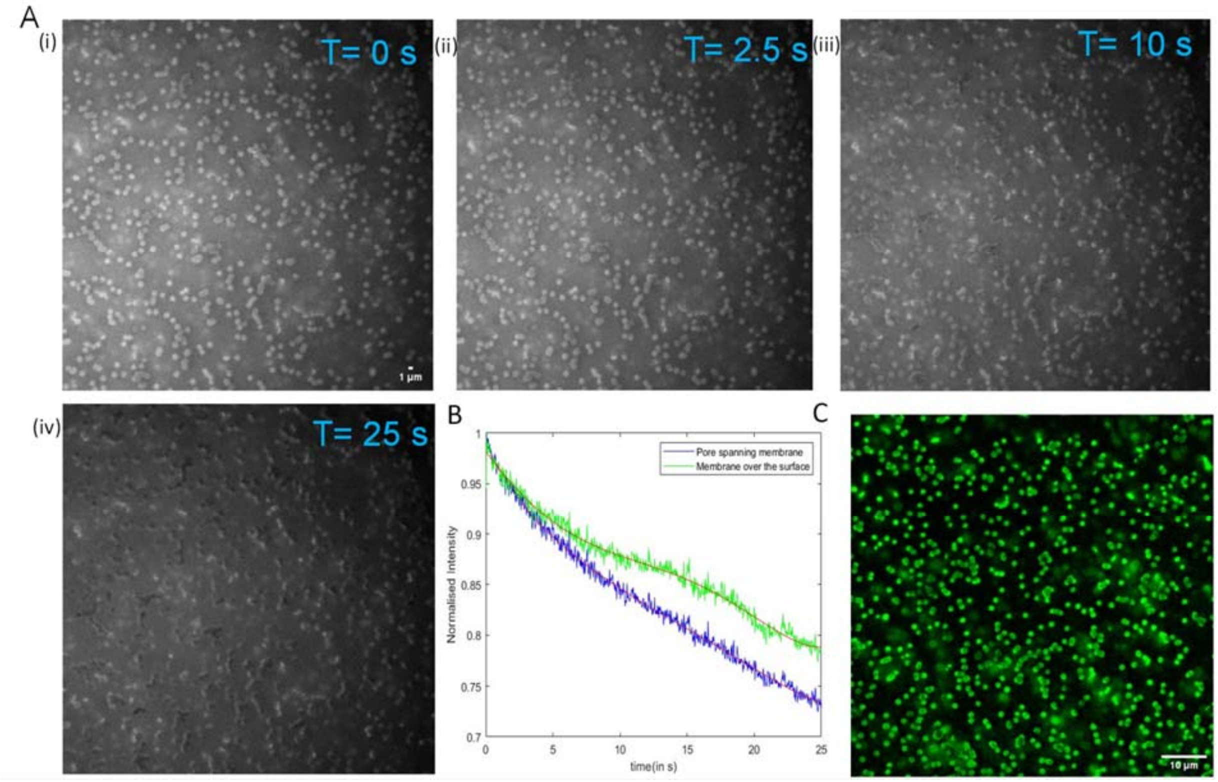

Figure S2: A. Photobleaching of the pore spanning membrane at time (i) 0 sec (ii) 2.5 sec (iii) 10 sec (iii) at 25 sec. The pore spanning membrane at 25 s is the indication of difference in mobility of the suspended and supported part of the pore spanning membrane. B. Variation of the normalized intensity over time for pore spanning suspended lipid bilayer membrane (Blue) and Supported lipid bilayer membrane (Green). C. 100 nM SRB entrapped PCTE SULB composed of POPC:CHOL (1:1). We used here 1 µm microwell sized substrate for this dye entrapped SULB preparation.

A B

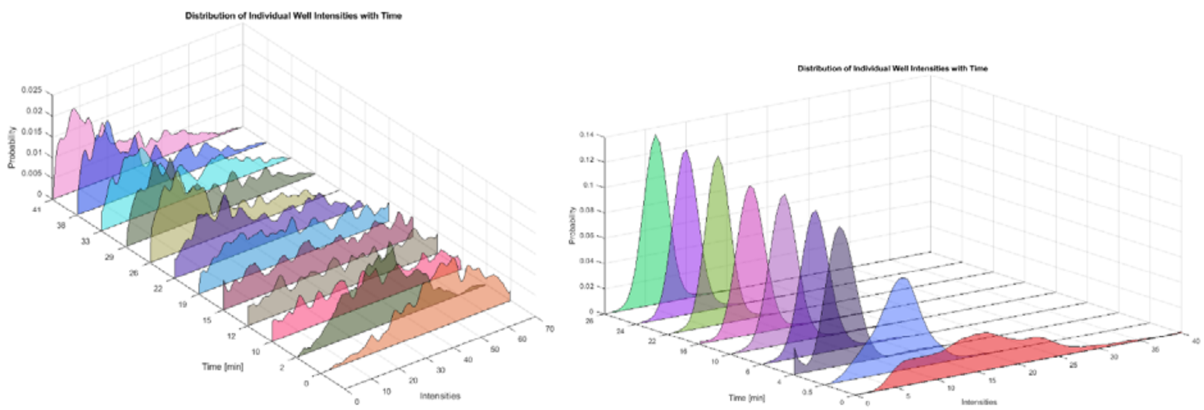

Figure S3: 2D histogram plots of temporal probability distribution of individual well intensity after addition of ClyA to the SULB system for (A) POPC:CHOL (1:1) and (B) POPC:SM:CHOL (1:1:1).It is to note that each SULB system is of 1 μm microwell size.

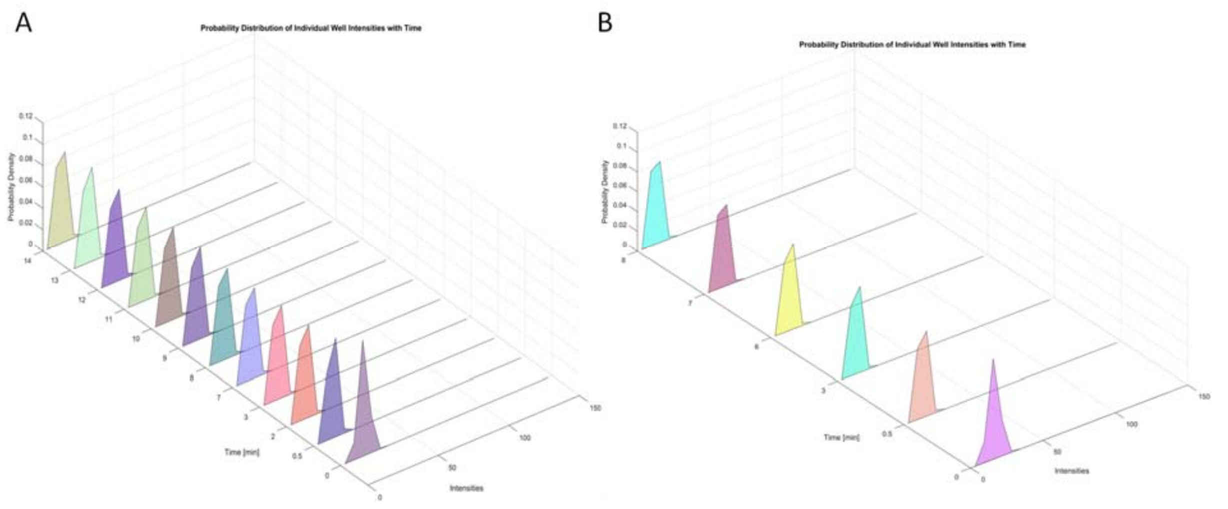

Figure S4: 2D histogram plots of temporal probability distribution of individual well intensity after addition of ClyA to the SULB system for (A) POPC:CHOL (1:1) and (B) POPC:SM:CHOL (1:1:1).It is to note that each SULB system is of 600 nm microwell size.

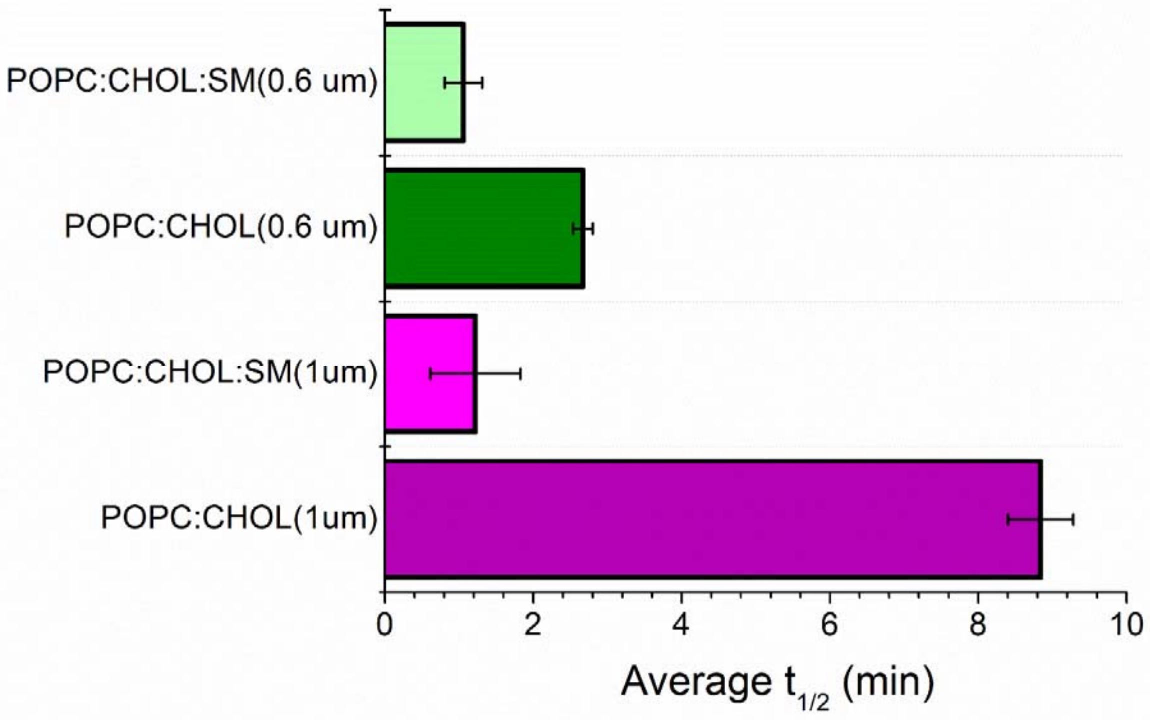

Figure S5: The half-lives for the pore-formation in SULB by Cly A

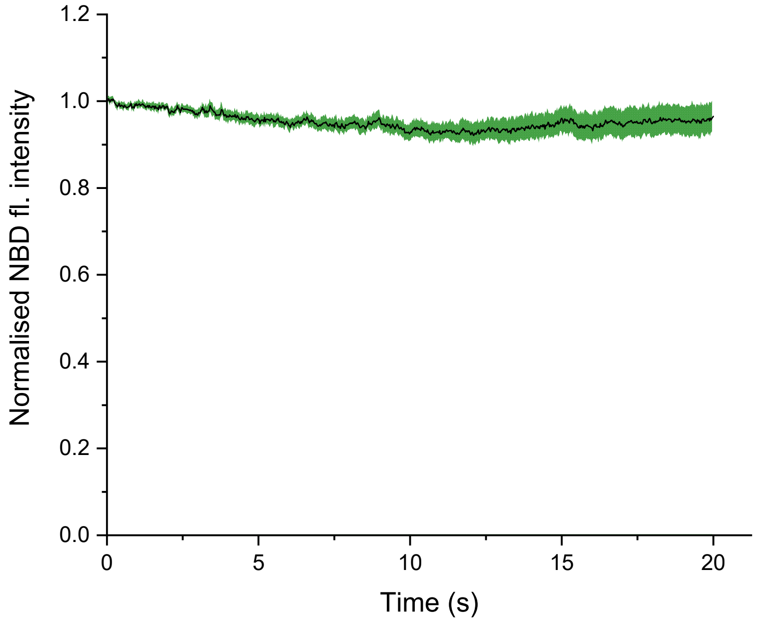

Figure S6: Plot of normalized NBD intensity with time in absence of ClyA and sodium dithionite.

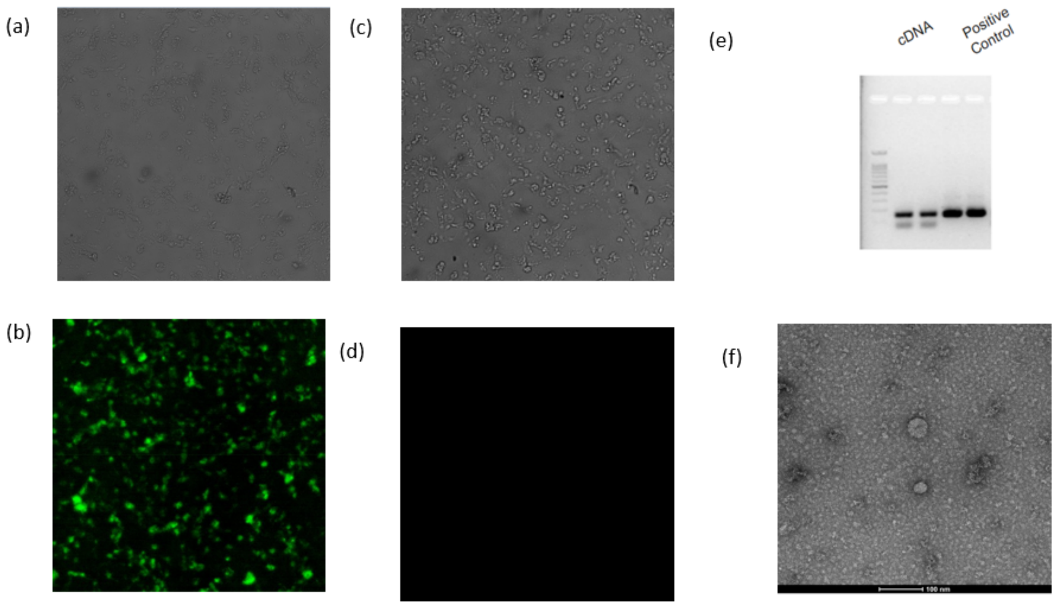

Figure S7: Characterization of Dengue virus. (a,b) the brightfield and immune fluorescent image of the Vero E6 cells after 72 hours of infection followed by immune-cytochemistry with anti-envelope antibody. (c,d) the brightfield and immunofluorescent images of uninfected Vero E6 cells followed by the same. (e) Gel image of the PCR product from the cDNA from the RNA extracted from the virus and original plasmid as positive control against the prM region. (f) Electron microscopic image of the dengue virus.
